# Molecular Organization Induced Anisotropic Properties of Perylene – Silica Hybrid Nanoparticles

**DOI:** 10.1038/s41598-017-07892-4

**Published:** 2017-08-10

**Authors:** Deepa Sriramulu, Shuvan Prashant Turaga, Andrew Anthony Bettiol, Suresh Valiyaveettil

**Affiliations:** 1 Department of Chemistry, National University of Singapore, 3 Science Drive 3, Singapore, 117543 Singapore; 2 Department of Physics, National University of Singapore, 2 Science Drive 3, Singapore, 117542 Singapore

## Abstract

Optically active silica nanoparticles are interesting owing to high stability and easy accessibility. Unlike previous reports on dye loaded silica particles, here we address an important question on how optical properties are dependent on the aggregation-induced segregation of perylene molecules inside and outside the silica nanoparticles. Three differentially functionalized fluorescent perylene - silica hybrid nanoparticles are prepared from appropriate ratios of perylene derivatives and tetraethyl orthosilicate (TEOS) and investigated the structure property correlation (**P-ST**, **P-NP** and **P-SF**). The particles differ from each other on the distribution, organization and intermolecular interaction of perylene inside or outside the silica matrix. Structure and morphology of all hybrid nanoparticles were characterized using a range of techniques such as electron microscope, optical spectroscopic measurements and thermal analysis. The organizations of perylene in three different silica nanoparticles were explored using steady-state fluorescence, fluorescence anisotropy, lifetime measurements and solid state polarized spectroscopic studies. The interactions and changes in optical properties of the silica nanoparticles in presence of different amines were tested and quantified both in solution and in vapor phase using fluorescence quenching studies. The synthesized materials can be regenerated after washing with water and reused for sensing of amines.

## Introduction

In recent years, fluorescent nanomaterials are used in optoelectronics, chemical sensing and bio-imaging applications^1, 2^. Owing to high mechanical and chemical stabilities, ease of functionalization and optical transparency make silica materials an ideal lattice for protecting and encapsulating fluorophores^3, 4^. Similarly, perylenediimide dyes are widely used as pigments, fluorescent sensors, and n-type semiconductors in organic electronic industries owing to high photostability, interesting optical and electronic properties^5, 6^. A few supramolecular architectures of conjugated dyes with fascinating optical properties have been reported in the literature^7, 8^.

Recently, we synthesized bright fluorescent silica nanoparticles by incorporating fluorophore inside the silica matrix and used for printing applications^9–11^. Electronic interactions arising from the strong aggregation of fluorophores during the growth of nanoparticles causes fluorescence quenching, which is undesirable^12, 13^. The fluorophore molecules inside the nanoparticle lattice could be organized through chemical functionalization^14^ or dispersed randomly inside the matrix^15, 16^. Such control on arrangement and distribution of molecules inside the silica lattice is expected to show interesting optical properties. Fluorescent materials provide a simple, sensitive and expedient method for detecting and sensing of amines^17, 18^. Recently, perylenediimide derivatives incorporated nanofibers were reported as fluorescent sensors for organic amine vapors^19–22^.

**Figure 1:**
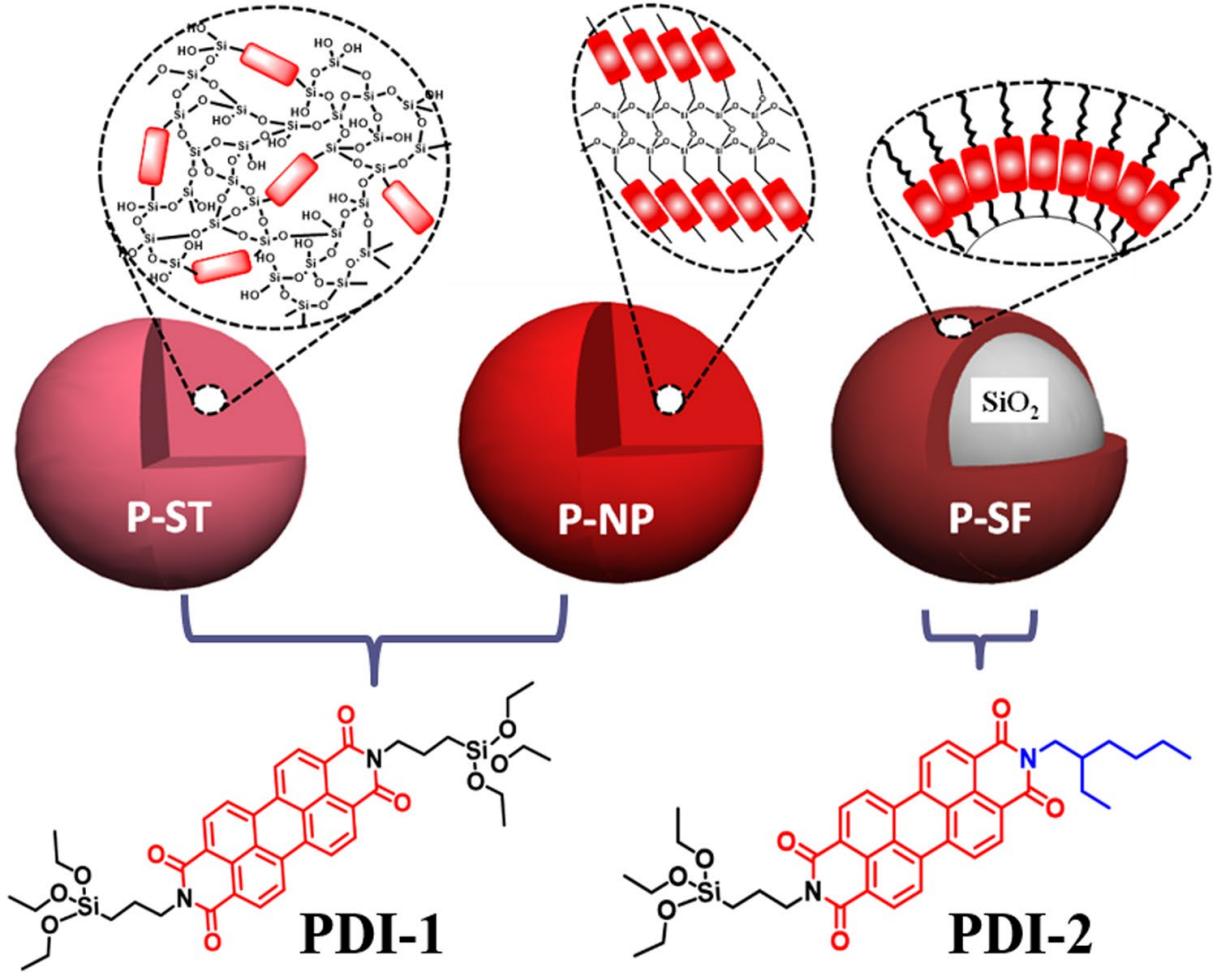
Three models of perylene incorporated silica nanoparticles with different organization of perylene molecules and the molecular structure of perylene silanes used for the synthesis.

Unlike the reported dye incorporated silica particles, here we address an important issue of how molecular orientation of perylene derivatives inside the matrix controls the optical properties of the silica nanoparticles. For this purpose, three types of silica nanoparticles were synthesized with different orientations and distributions of perylene molecules. First type of particles was synthesized using Stoeber method^23^ which gave monodispersed spherical particles with homogeneous, but random distribution of perylene molecules inside the silica matrix (**P-ST**). In the second type, homopolymerization of perylene disilane precursor was used for the preparation of silica nanoparticles (**P-NP**). In the third particle, perylene was incorporated on the surface of silica nanoparticles through chemical immobilization (**P-SF**). During this investigation, the distribution and organization of perylene molecules inside the silica matrix were controlled via appropriate synthetic route employed (Fig. 1). The observed optical properties of the particles are correlated with the orientation of perylene molecules inside the silica matrix. Such differentially functionalized perylene-silica nanoparticles are able to interact with electron rich amines and show interesting anisotropic optical properties.

## Results and Discussion

### Design strategy, synthesis and characterization of perylene silica nanoparticles

**Figure 2:**
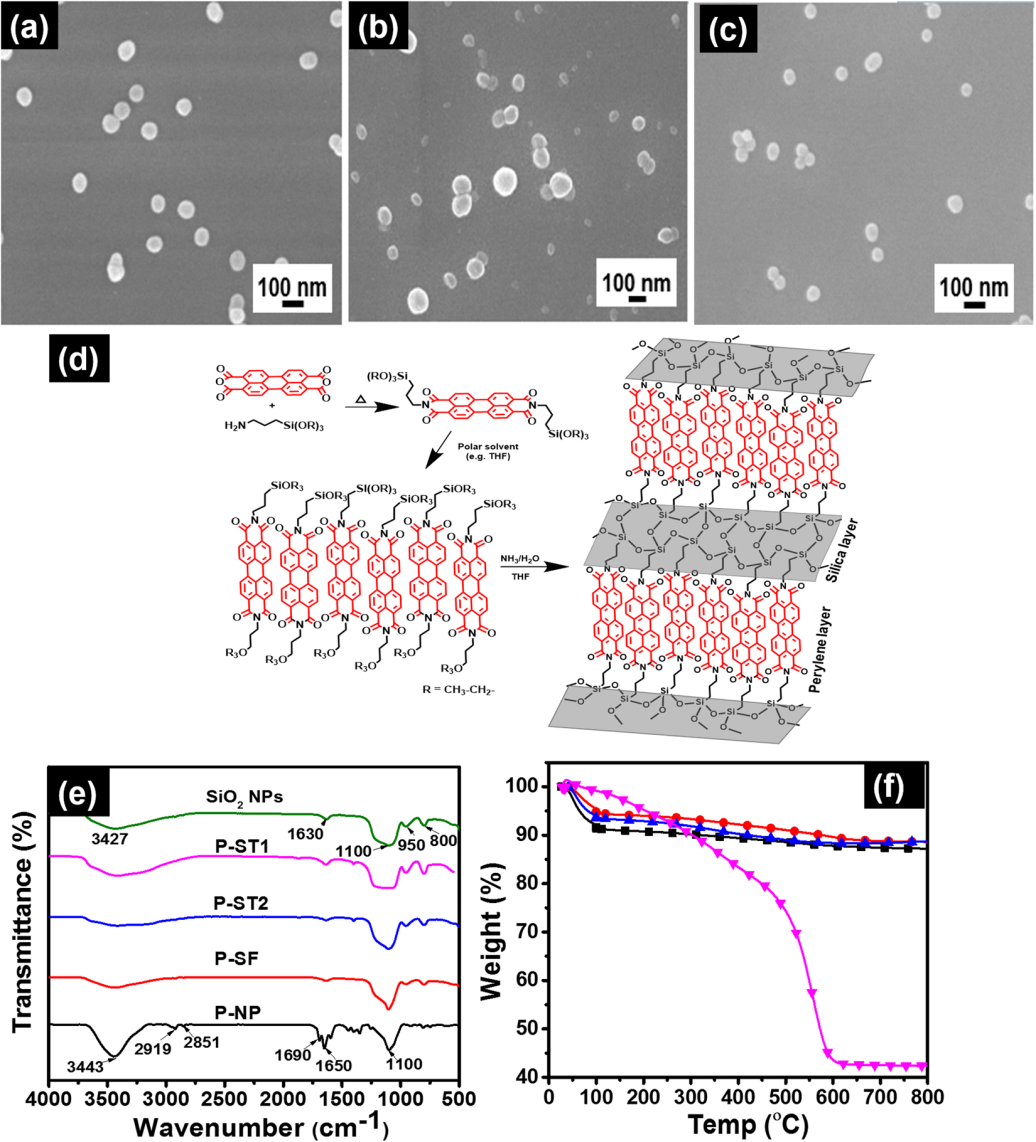
SEM morphologies of **P-ST** (**a**), **P-NP** (**b**) and **P-SF** (**c**). Polymerization of PDI-1 (**d**) to form perylene rich region and silica region. FTIR spectra (**e**) of unmodified silica NPs, **P-ST1**, **P-ST2**, **P-SF** and **P-NP**. TGA traces (**f**) of **P-ST1** (■), **P-ST2** (), **P-SF** (), and **P-NP** () particles in air.

Perylene silica nanoparticles (**P-ST**, **P-NP**, and **P-SF**) were synthesized using PDI-1 and PDI-2 as silane precursor (Fig. 1). PDI-1 and PDI-2 were prepared using known procedures by reacting perylene derivatives with aminopropyltriethoxysilane under appropriate conditions (Supplementary methods S1, S2)^24–26^. **P-ST** nanoparticles were prepared using a modified Stoeber method (Supplementary method S3) with TEOS as silica precursor and ammonia as catalyst in ethanol solvent. The base catalyzed hydrolysis of TEOS molecules resulted in Si-O-Si linkages of the silica network. Presence of bispropyltriethoxysilyl moiety of PDI-1 silane precursor can be used to attach perylene to the silica network. The prepared perylene silica nanoparticles of around 300–100 nm in size were labeled as **P-ST1** (0.010 g), **P-ST2** (0.080 g) depending on the initial concentration of perylene silane precursor used in the synthesis (Fig. 2a) and also on the composition of ethanol, ammonia and water. After increasing the molar ratio of PDI-1 and TEOS to 0.5, the experimental conditions used for the synthesis of **P-ST1** particles yielded polydispersed agglomerated **P-ST2** nanoparticles. In order to obtain monodispersed spherical **P-ST2** particles, we changed the experimental conditions (Supplementary Table S1).

**P-NP** nanoparticles were synthesized using nanoprecipitation method, which involves mixing of reagents in two miscible solvents such as water and THF (Supplementary method S4). Appropriate amounts of the PDI-1 precursor dissolved in THF were mixed with aqueous ammonia solution to start polymerization. Polymerization was allowed to run at room temperature and the mixture was lyophilized to get perylene nanoparticles with an average size of ~100 nm (Fig. 2b). Triethoxysilyl groups on each end of the perylene diimide are polymerized to form siloxane network, thereby organizing perylene inside the silica matrix (Fig. 2d). In addition, the aggregation tendency of perylene also helps to achieve higher molecular order inside the lattice.

The **P-SF** nanoparticles were prepared using surface functionalization of bare silica nanoparticles with PDI-2 (Supplementary method S5). Bare silica nanoparticles were prepared using reverse microemulsion method to yield monodispersed particles of size around ~50 nm, followed by surface modification using chemical immobilization of differentially functionalized PDI-2 though triethoxysilyl group to form **P-SF** nanoparticles (Fig. 2c).

The particle size and zeta potential of all the particles were measured using zetasizer instrument (Supplementary Table S2). All the particles gave large hydrodynamic diameter compared to SEM images, and larger negative zeta potential (~ −30 to −40 mV).

The FT-IR spectra of pristine and modified silica nanoparticles (bare SiO_2_, **P-ST1**, **P-ST2**, **P-SF** and **P-NP**) are shown in Fig. 2e. For unmodified silica nanoparticles, the peak observed at 3427 and 1630 cm^−1^ corresponds to vibrations of Si-OH in silica. The intense band at 1100 cm^−1^ is assigned to Si-O-Si asymmetric stretching vibration^27^, whereas the Si-OH vibration bands are observed at 950 and 800 cm^−1^. **P-NP** nanoparticles showed prominent peaks at 1690 and 1650 cm^−1^ corresponding to imide C=O stretching, 1595 cm^−1^ for aromatic C=C stretching, 2919–2851 cm^−1^ (C-H stretching of alkyl chains) in addition to peaks corresponding to silica. As expected, peaks associated with functional groups on perylene units showed higher intensity in **P-NP** system than **P-ST2**, owing to the high concentration of perylene encapsulated inside the nanoparticle matrix.

Thermogravimetric analyses (TGA) of silica particles were performed using a heating rate of 10 deg/min in air atmosphere from 40 °C to 800 °C (Fig. 2e, DTA of silica particles are available in Supplementary Fig. S1) and the results are tabulated in Supplementary Table S3. All particles showed first weight loss of about 5–8% below 150 °C, which is assigned to the loss of adsorbed water (Fig. 2f)^28^. The second weight loss observed from 150 °C – 400 °C is due to decomposition of aliphatic chains attached to perylene and unreacted ethoxy groups on silane functional group. Third region of weight loss observed from 400 °C–800 °C is explained as complete degradation of perylene core units and further dehydration of silica matrix. As expected, significant weight loss (~55%) was observed for **P-NP** nanoparticles in the region of 150 °C–800 °C due to the complete degradation of large amounts of perylene derivatives incorporated inside the particles. The weights loss observed was correlated to the theoretical amount of perylene present in silica nanoparticles as tabulated in Supplementary Table S4. **P-NP** nanoparticles after calcinations at 850 °C under nitrogen atmosphere showed traces of charred carbon residues. All other silica nanoparticles showed white powder at the end of calcinations (Supplementary Fig. S2).

### Photophysical properties of fluorescent silica nanoparticles

**Figure 3:**
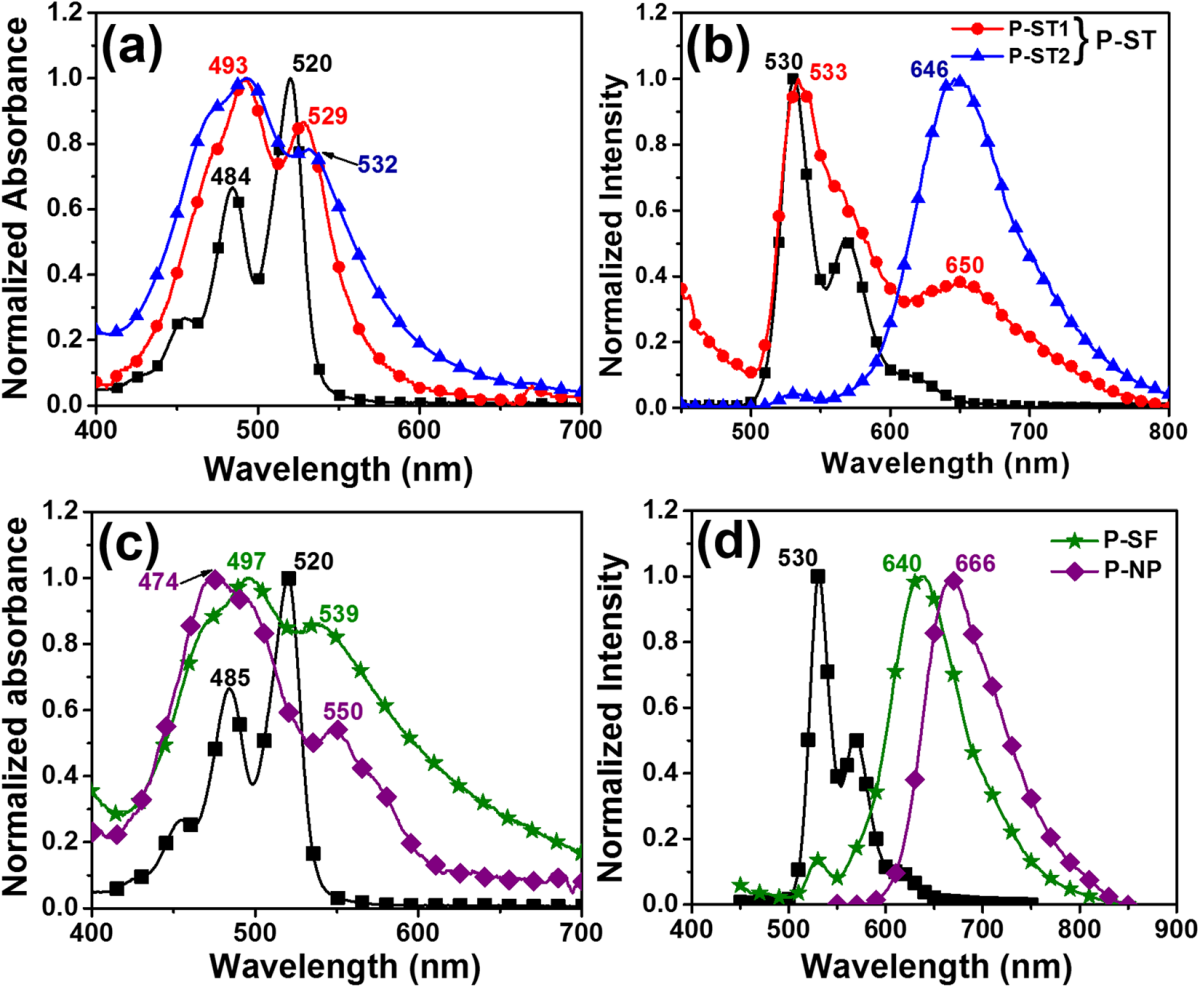
Absorbance (**a,c**) and emission spectra (**b,d**) of (■) PDI silane monomer, **P-ST1**(), **P-ST2**(), **P-SF** () dispersed in THF and **P-NP** () dispersed in water. Excitation wavelength was 350 nm.

The absorption and emission spectra of PDI-1 silane monomer dissolved in THF (Fig. 3a and b), showed typical characteristic monomeric features of perylene absorption (460 nm, 480 nm and 520 nm) and emission (530 nm, 570 nm and 620 nm) peaks in solution which corresponds to 0–2, 0–1 and 0–0 electronic transitions^29, 30^. The photophysical properties of perylene silica nanoparticles (P-ST, **P-SF**, **P-NP**) were investigated using spectrophotometric methods (Fig. 3) and summarized in Table 1. The prepared nanosilica particles showed changes in absorbance and emission properties based on the concentration and orientation of perylene molecules. All three series of silica nanoparticles showed an increase in intensity of (0–1) vibronic band with respect to 0–0 transition. This is typically observed for PDI chromophores stacked in H-type aggregates, where the fluorophores are cofacially arranged^31^. The ratio of intensities of the (0–0) and (0–1) transitions is often used as an indication to measure extent of aggregation of PDI chromophores^32, 33^. The absorbance ratio of two transitions (0–0/0–1) is 1.66 for monomeric PDI silane compounds in solution, which indicates free PDI molecules are dispersed individually with no aggregate formation in solution.

**Table 1:** Summary of photophysical properties of perylene silica nanoparticles.

Perylene silica NPs	λ_max_, nm	λ_emi_, nm	Absorbance peaks ratio^a)^	Φ, quantum yield (%)	Life time (τ, ns)	Dichroic ratio (D)	Order parameter (R)
P-ST1	(A_0–1_) 492, (A_0–0_) 529	533, 650	0.86	6	4.35	**—**	**—**
**P-ST2**	(A_0–1_) 493, (A_0–0_) 532	646	0.78	3.6	4.02	1.3	0.09
**P-NP**	(A_0–2_) 474, (A_0–1_) 494, (A_0–0_) 550	666	0.54	1.8	5.38	4.2	0.5
**P-SF**	(A_0–1_) 497, (A_0–0_) 539	640	0.85	4.7	3.38	1.4	0.1
PDI precursor	(A_0–1_) 485, (A_0–0_) 520	530	1.51	70	3.33	**—**	**—**

^a)^ Peak-to-peak ratio of the absorbance at (0, 0)/(0,1).

In the case of P-ST particles, incorporation of PDI into silica network led to an increase in the intensity of 0–1 transition when compared to 0–0 transition and the ratio of absorbance from these two transitions (0–0 / 0–1) was found to be 0.8, which indicates significant aggregation of PDI inside the silica matrix (Fig. 3a). By varying the amount of PDI concentration (**P-ST1**, **P-ST2**) inside the silica matrix, changes in emission maxima were observed as compared to PDI silane precursor in THF solution (Fig. 3b). **P-ST1** particles showed two emission maxima, one at 533 nm corresponds to monomeric PDI peak and another broad peak at 652 nm from aggregated PDI molecules inside silica matrix (Fig. 3b). Upon increasing the concentration of PDI in **P-ST2**, more prominent single excimer peak was observed at 646 nm, due to stronger *pi-pi* interaction and formation of well-organized molecular aggregates^34^.

Similarly, **P-NP** silica nanoparticles formed via nanoprecipitation method showed interesting optical behavior when compared to P-ST and **P-SF** particles. Absorption spectrum of **P-NP** showed an increase in intensity of 0–2, 0–1 than 0–0 transition peak (Fig. 3c, Table 1). The absorbance ratio for the two transitions 0–0/0–1 is 0.5, which indicates high degree of ordering or packing of perylene molecules inside the nanoparticles. Emission maximum for **P-NP** was observed at 666 nm, with a red shift of 16 nm as compared to **P-SF** particles (640 nm, Fig. 3d).

Interestingly, **P-SF** particles showed absorption spectrum similar to P-ST nanoparticles. Absorption intensity ratio A_0–0_/A_0–1_ of **P-SF** is around 0.9, which implies the presence of a few perylene aggregates (Fig. 3c). The emission spectrum of **P-SF** showed an excimer maximum around 640 nm and small shoulder peak around 530 nm indicating the presence of perylene aggregates on the surface of silica nanoparticles (Fig. 3d). Photophysical properties of all perylene-silica nanoparticles showed significant changes in absorption and emission spectra depending on the concentration and organization of perylene molecules inside the silica lattice (Table 1). As expected, excimeric peak around 640 nm become predominant with increase in the concentration of perylene molecules for all silica nanoparticles investigated.

### Fluorescence lifetime measurements

Besides absorption and emission spectra^35^, fluorescence quantum yield and fluorescence lifetime of particles were also measured and summarized in Table 1 ^35^. Decrease in quantum yield (Φ) was observed with increase in π-π stacking of perylene molecules in silica nanoparticles as shown for **P-ST1** (6%), **P-SF** (4.7%), **P-ST2** (3.6%) and **P-NP** (1.8%). Quantum yield of dilute solution (i.e. no aggregation) of PDI in THF is 70%, but once encapsulated in silica matrix quantum yield decreases due to the formation of nonemissive aggregates^23^. Fluorescence life time measurement indicates different chemical environment for perylene units inside the three types of silica nanoparticles. PDI dissolved in THF showed a lifetime of 3.33 ns and highest lifetime was observed for **P-NP** (5.38 ns) followed by **P-ST1** (4.35 ns), **P-ST2** (4.05 ns) and **P-SF** (3.38 ns). The observed increase in lifetime of **P-NP** and P-ST can be attributed to two reasons such as formation of H-aggregates^36^ and difference in chemical environment for perylene molecules inside the silica lattice.

### Steady state fluorescence anisotropy measurements

**Figure 4:**
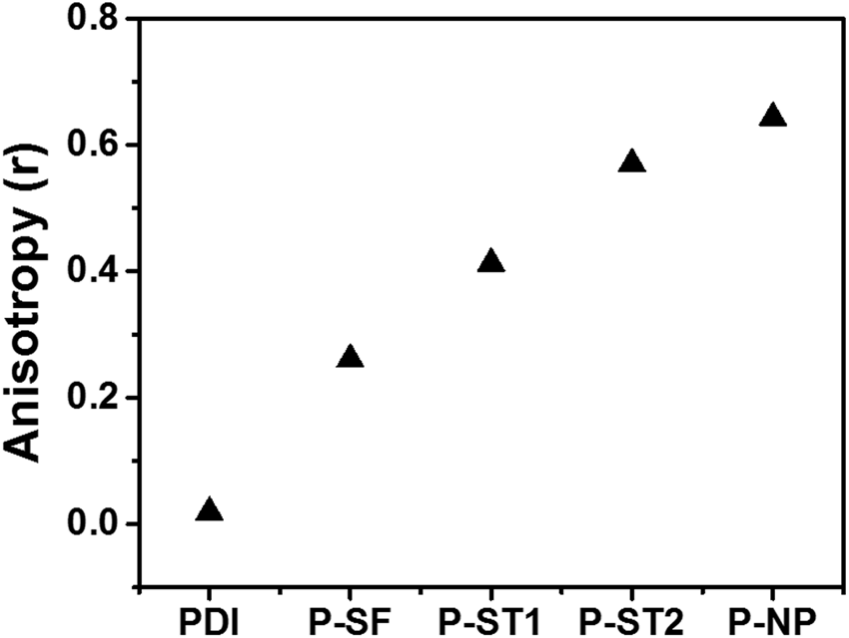
Fluorescence anisotropy (r) of PDI molecule, **P-SF**, **P-ST1**, **P-ST2** nanoparticles dispersed in THF and **P-NP** nanoparticles in water.

Organization dependent anisotropic nature on emission properties was investigated using silica nanoparticles to understand distribution of dyes inside or outside the silica nanoparticles (Fig. 4)^37, 38^. Fluorescence anisotropy (r) measures the degree of polarization of the emission obtained when a fluorophore was excited using a polarized light. For measuring anisotropy, samples were excited with vertically polarized light and emission intensity was measured. Emission intensities parallel (I_vv_) and perpendicular (I_vh_) with respect to polarization of the light used for excitation are recorded and used to calculate anisotropy (r) by using the Equation 1.

1 $${\rm{r}}=\frac{{{\rm{I}}}_{{\rm{vv}}}-{{\rm{I}}}_{{\rm{vh}}}}{{{\rm{I}}}_{{\rm{vv}}}+2{{\rm{I}}}_{{\rm{vh}}}}\,$$

Anisotropic measurements revealed the angular displacements of dye molecules that occur between the absorption and emission of a photon. This angular displacement is dependent on the rotational diffusion of dye molecules during the lifetime of the excited state. Perylene diimide (PDI) dissolved in THF solvent (i.e. completely disordered dynamic system) showed a lowest anisotropic effect (0.017) and **P-NP** (i.e. with high degree of ordering) gave the highest anisotropic value of 0.65. Such values can be explained using the restricted movement of the well-aligned arrangement of perylene molecules inside the silica matrix as compared to the random arrangement of molecules in solution. To differentiate the behavior of perylene molecules with respect to the mode of functionalization, the optical properties of **P-SF** nanoparticles with perylene molecules incorporated on the surface were compared with P-ST nanoparticles with random arrangements of the perylene molecules (Fig. 4). **P-SF** silica nanoparticles showed an anisotropic factor of ~0.25, but less than that of **P-ST2** (0.58) and **P-NP** (0.65) particles. PDI-2 functionalised on the surface of silica nanoparticles in **P-SF** are much more mobile than PDI-1 incorporated inside the other silica nanoparticles. Thus, observed anisotropic values confirm that PDI-1 units incorporated inside the silica particles (i.e. P-ST, **P-NP**) are fixed and not dynamic as compared to PDI-2 units in **P-SF**.

### Polarized absorbance measurements

**Figure 5:**
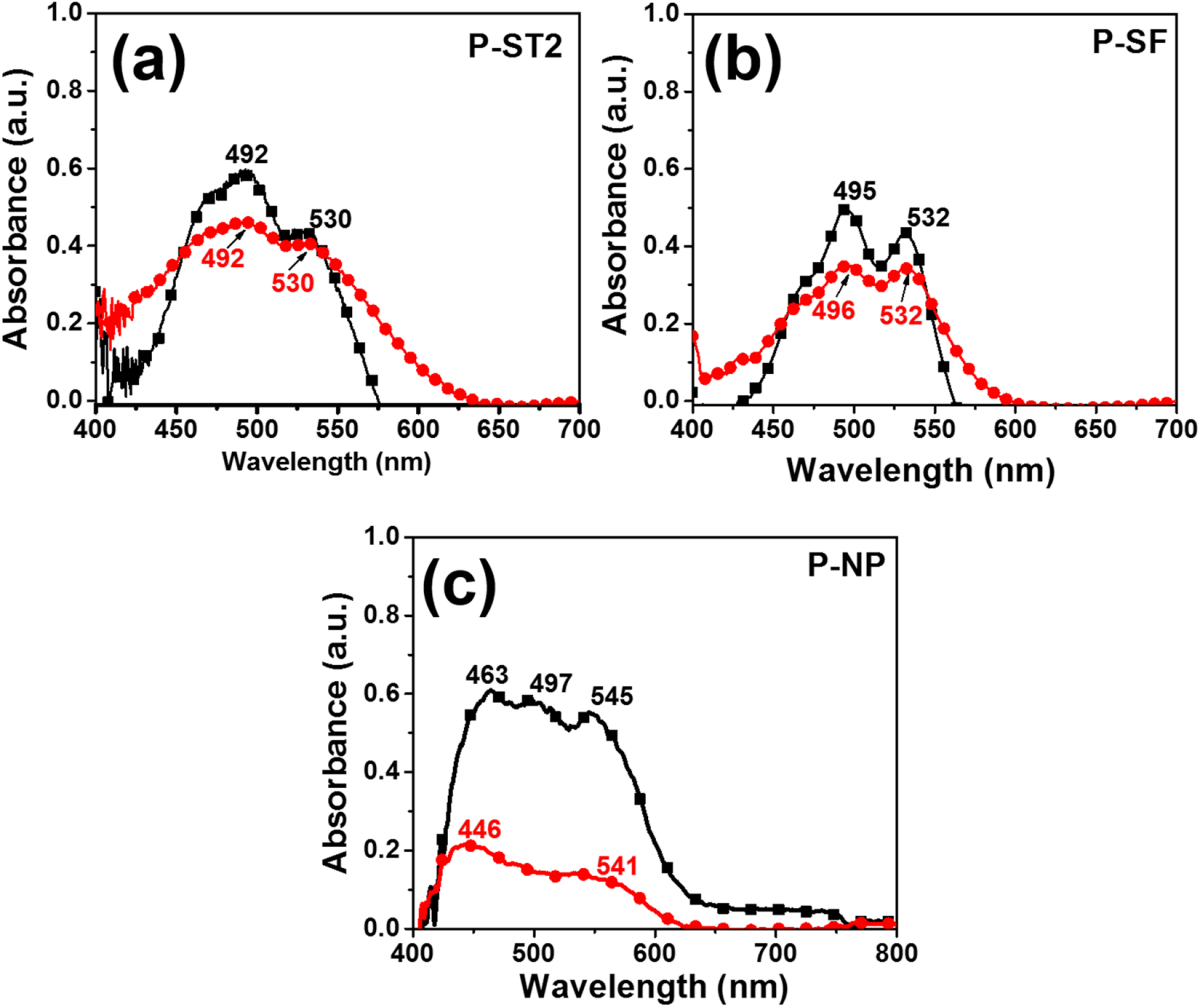
Polarized UV/Vis absorption spectra of perylene silica nanoparticles (**a**) **P-ST2**, (**b**) **P-SF** and (**c**) **P-NP** with the samples set parallel (||, ■) and perpendicular (⊥, ) to the polarizer.

The alignment of dye molecules in nanoparticles can be measured using solid state polarized UV-Vis spectroscopy (Fig. 5, Table 1)^39, 40^. The experimental dichroic ratio (D) of the dye is defined as the ratio of the absorbance of the dye in nanoparticles oriented parallel (A_‖_) and perpendicular (A_⊥_) to the incident polarized light as shown in Equation (2)^41^. The observed dichroic ratios are used to measure the order parameter or anisotropic measurement of the dye (R), as shown in Equation (3)^42^. Anisotropic value of 1 indicates that dye molecules are fully aligned and a value of 0 indicates molecules have no alignment.

2 $$D=\frac{{A}_{\parallel }}{{A}_{\perp }}$$

3 $$R=\frac{D-1}{D+2}$$

Polarized absorption spectra revealed that, **P-NP** nanoparticles gave a larger dichroic ratio of D = 4.2, from which order parameter (R) of 0.5 was obtained. **P-ST2** and **P-SF** nanoparticles gave a dichroic ratio (D) of about 1.3 and 1.4, respectively, with order parameter of about 0.09 for **P-ST2** and 0.1 for **P-SF** nanoparticles. **P-NP** nanoparticles showed high degree of anisotropy (R = 0.5) when compared to **P-ST2** and **P-SF** nanoparticles, indicating molecules are aligned inside **P-NP** nanoparticles and randomly dispersed in **P-ST2** and **P-SF**.

### Amine sensing properties

Perylene derivatives were explored for sensing amine vapors in solid state and in solution^43^ due to electron deficient nature, high photostability and solid state emission properties. Perylene incorporated silica nanoparticles were used to investigate the interaction with structurally different amines such as butylamine, triethylamine, diisopropylethylamine and aniline. Percentage quenching efficiency was calculated from the fluorescence data using the Equation (4).

**Figure 6:**
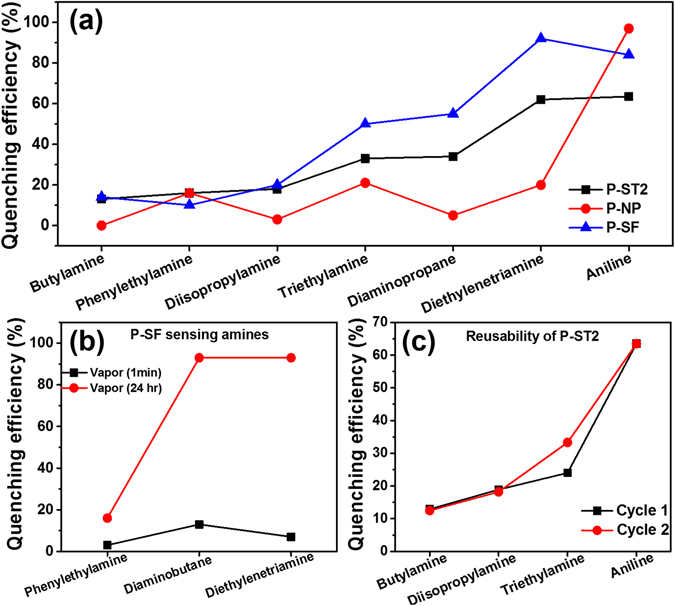
Percentage quenching efficiency of **P-ST2**, **P-SF** and **P-NP** nanoparticles in sensing (**a**) butylamine, diisopropylamine, triethylamine, aniline and biogenic amines such as phenylethylamine, diaminopropane and diethylenetriamine in solution. (**b**) **P-SF** nanoparticles sensing biogenic amines both in solution (~1 mins) and vapor phase (24 hr). (**c**) Reusability of **P-ST2** particles for sensing amines in THF solution. Excitation wavelength was 350 nm.

As expected, **P-SF** showed higher quenching efficiency to amines when compared to **P-ST1** and **P-ST2** in solution (Fig. 6a) and spectra are given in the supporting information (Supplementary Figs S3–S5). Such response indicates that only perylene present on the surface of the silica are available for sensing of amines. The quenching mechanism can be attributed to photoinduced electron transfer from HOMO of electron donor amines to HOMO of excited state of electron deficient PDI molecules. Aniline showed higher quenching efficiency than other amines due to favorable energy difference between HOMO of aniline (−5.39 eV) and HOMO of PDI (−5.99 eV), which facilities faster electron transfer from aniline to PDI molecules^20^. Interestingly, **P-NP** particles showed 96% quenching efficiency over other particles, this could be due to enhanced exciton migration via intermolecular π-π interactions, indicating well organized packing of perylene molecules inside the **P-NP** nanoparticles, enabling amplified fluorescence quenching by surface adsorbed aniline molecules^44^. Similarly, these nanoparticles were also used as fluorescent probe for sensing biogenic amines such as phenylethylamine (PEA), diaminopropane (DAP) and diethylenenetriamine (DETA) in solution (Fig. 6a and Supplementary Fig. S6). Quenching efficiency for DETA was highest when compared to DAP and PEA for all three silica particles (**P-ST2**, **P-SF**, **P-NP**).

As expected, **P-SF** nanoparticles showed highest quenching efficiency for DETA (98%) as compared to **P-ST2** (60%) and **P-NP** (20%). Functionalized **P-SF** nanoparticles showed higher quenching efficiencies when compared to P-STs and **P-NP**s, as most of the perylene molecules distributed within silica matrix are not accessible for amines. **P-SF** showed higher quenching efficiency for DETA due to more favorable energy difference between HOMO of DETA and HOMO of perylene molecules. The driving force for photoinduced electron transfer is high for DETA, which facilitates efficient fluorescent quenching in solution. This indicates that **P-SF** silica nanoparticles can selectively sense DETA in presence of other amines.

Optical photographs of the particles dropcasted on a coverslip before and after exposure to the amines vapors showed visible color changes owing to electron transfer quenching process (see Supplementary Fig. S7). All particles showed high quenching efficiency towards amines after 24 hrs exposure in vapor state (summarized in Supplementary Fig. S8). Figure 6b showed the comparison of **P-SF** particles towards sensing of amines vapors for 1 min and 24 hr. For example, quenching efficiency for **P-SF** nanoparticles in presence of DETA was 92% in solution phase (Fig. 6a) and just 10% in vapor phase (exposure time to amine in both cases was 1 mins). Accordingly, DETA – perylene particle interaction/quenching in THF is more effective than in vapor phase, as the quencher (amine) strongly interact with perylene on the surface of the particles in solution.

As a proof of concept, reusability of perylene silica nanoparticles were explored after washing of nanoparticles with THF and drying them in an oven at 70 °C for 24 hours (Fig. 6c, Table 2). Perylene silica nanoparticles showed consistent quenching efficiencies after repeated cycles and indicated that such nanoparticles can be used for sensing of amines.

**Table 2:** Percentage quenching efficiency and reusability of perylene silica nanoparticles in sensing amines in solution.

Amines	P-ST1		P-ST2		P-NP	
	Cycle 1	Cycle 2	Cycle 1	Cycle 2	Cycle 1	Cycle 2
Triethylamine	42.9%	42.8%	33.3%	24%	21%	11%
Aniline	52%	45.5%	63.5%	63.5%	97%	94%

## Conclusions

Here we report the synthesis and characterization of three optically anisotropic silica nanoparticles with significant differences in the organization of perylene molecules inside the silica matrix. In the P-ST particles, perylene derivatives were randomly distributed and organized inside the silica lattice by varying the composition of the starting materials and random copolymerization of perylene silanes with TEOS. In the **P-NP** particles, a bifunctionalized perylene silane was homopolymerized to accommodate *pi-pi* stacking induced organization inside the silica lattice and in **P-SF**, prepared silica particles are post functionalized using monofunctional perylene silane on the surface. Structural organization of the perylene molecules inside the silica nanoparticles was established using UV-Vis absorption spectroscopy, emission spectroscopy, fluorescence anisotropy, lifetime measurements, polarized spectroscopy studies and solid state absorption anisotropy. **P-NP** showed a high degree of anisotropy and a red shift in excimeric peak at 666 nm as compared to P-ST (646 nm) and **P-SF** (640 nm) nanoparticles indicating higher degree of organization and H-aggregate formation within the **P-NP** nanoparticles. All silica nanoparticles were explored for detecting amines in solution and in vapor phase. As expected, **P-SF** and **P-NP** particles showed highest quenching efficiency for aniline (~80–90%) when compared to P-ST nanoparticles, owing to the easy access of electron deficient perylene for the amines. All nanoparticles can be efficiently regenerated and reused, without compromising on sensing ability of amines.

## Methods and Materials

### Materials

Tetraethylorthosilicate (TEOS), 3-aminopropyltriethoxysilane (APTES), absolute ethanol, ammonium hydroxide solution (25%), were purchased from Aldrich. All solvents and reagents (analytical grade and spectroscopic grade) were obtained from commercial sources and used as a received.

### Characterization Methods

^1^H Nuclear magnetic resonance (NMR) spectra were recorded on Bruker Avance AV300 (300 MHz) NMR instrument using CDCl_3_ as the solvent. The IR spectra were recorded in the range of 4000–400 cm^−1^ using a Bruker ALPHA FT-IR Spectrophotometer with a resolution of 4.0 cm^−1^. Particle morphology was examined using scanning electron microscopy (JEOL JSM-6701F). The UV/Vis absorption studies were performed on a Shimadzu-1601 PC spectrophotometer. The steady state fluorescence studies were carried out on an Agilent Cary Eclipse Fluorescence Spectrophotometer.

For quantum yield measurement, following Equation (4) was used to measure relative quantum yield of particles.

4 $${\Phi }={{\Phi }}_{R}{\rm{x}}\frac{I}{{I}_{R}}x\,\frac{{A}_{R}}{A}{\rm{x}}\frac{{\eta }^{2}}{{\eta }_{R}^{2}}$$

where, *Φ*_*R*_ *, I*_*R*_ *, A*_*R*_ and *η*_*R*_ refers to quantum yield, integrated area under emission spectra, absorbance and refractive index of reference, Rhodamine B (*Φ* *R*  =  0.7 and *η* = 1.36) in ethanol^45^ solvent, *I, A* and η refers to integrated area under emission spectra, absorbance and refractive index of sample in THF solvent.

### Fluorescence Quenching Study of Perylene Silica Nanoparticles for Amine Sensing

For sensing amines in solution, 0.2 mmol of amines (butylamine, diisopropylamine, triethylamine, aniline, phenylethylamine, diaminobutane (putriscine) and diethylenetriamine) were added into a dispersion of perylene silica nanoparticles (**P-ST2** or **P-SF1**) in THF solvent (2 mL) or **P-NP** dispersed in water (2 mL). The emission spectrum was recorded before and after adding amines to measure changes in fluorescence and efficiency of fluorescence quenching was calculated using the equation (5) given below.

5 $${\rm{Quenching}}\,{\rm{efficiency}}( \% )=(\frac{{{\rm{I}}}_{{\rm{o}}}-{I}_{}}{{{\rm{I}}}_{{\rm{o}}}})\,{\rm{x}}\,100\,$$

where, I_o_ and I are the fluorescence intensities of perylene silica nanoparticles in the absence and presence of amines, respectively.

Sensing of amines using solid films of particles were performed as follows. Thin films of perylene silica nanoparticles (2.5 mg) dispersed in solvents such as THF or water were cast on quartz plate and dried in an oven at 70 °C for a day for complete removal of solvent. Quartz plate containing perylene silica nanoparticles were exposed to amine vapors for a known period (1 min or 24 hrs). Fluorescence spectra were recorded before and after exposure to amines.

### Absorption Anisotropy Measurement

**Figure 7:**
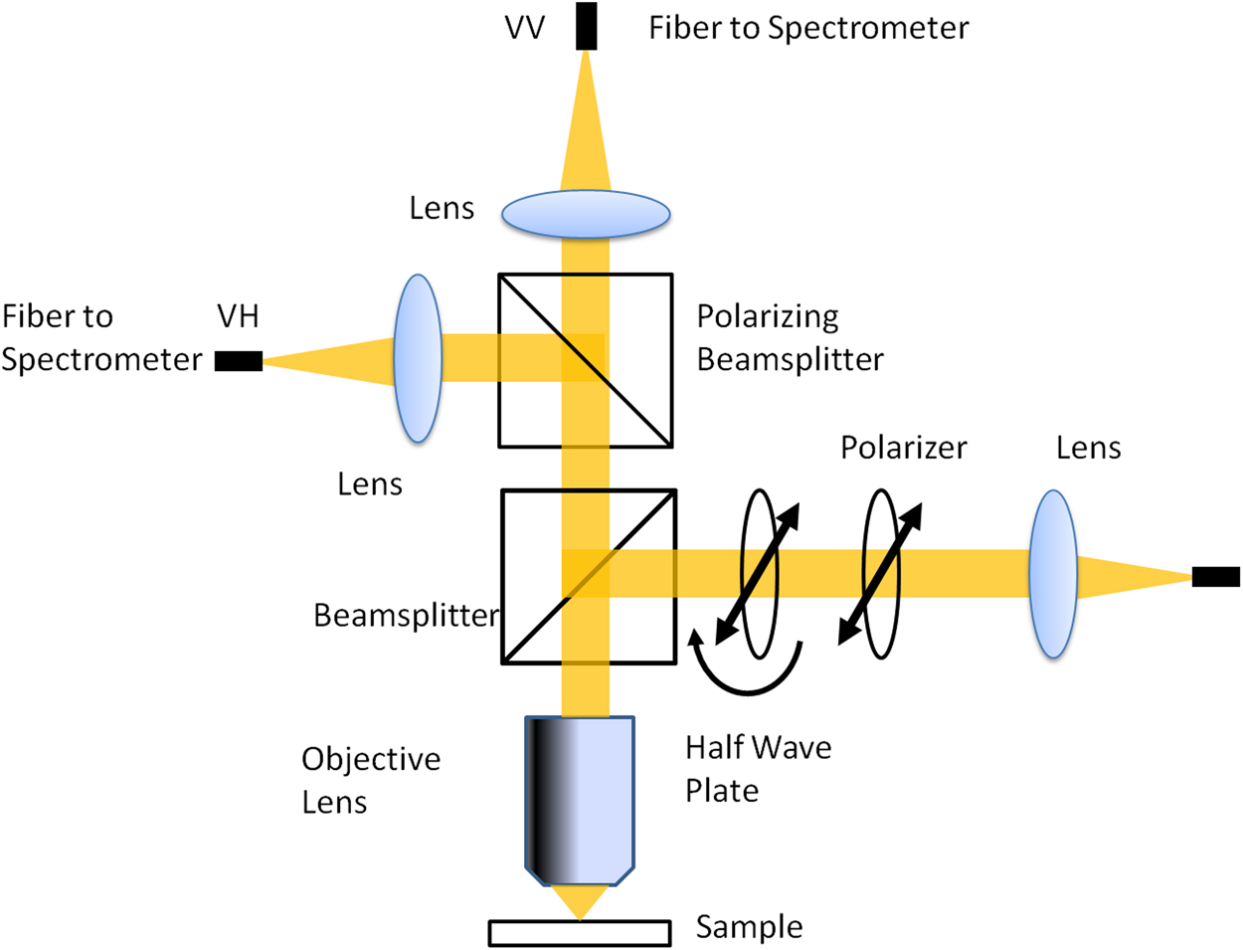
Schematic representation of solid state absorption anisotropy setup.

For absorption anisotropy studies, a custom microscope attached with a polarizer and an analyzer was constructed. The light source used involves both halogen and deuterium lamps (Ocean Optics D-2000 BAL) with a wavelength range from 250 to 2500 nm. Figure 7 shows the details of the absorption anisotropy setup. The input light comes through a fibre of about 600 µm core diameter and the light is collimated using a lens. After collimation, the light is passed through a polarizer and a half-wave plate to control the incident light polarization. The light is focused on the sample with a 40x objective. The back-reflected light was passed through a polarization analyser and finally collected into the optical fibre connected to the UV-Vis Spectrophotometer (Ocean Optics Q65000).

Our measurement range is limited to the polarizer working spectral range from 420 to 680 nm. The samples were mounted on a Teflon substrate and the background signal was measured using blank quartz substrates. Samples were prepared by dropcasting a thin film of nanoparticles dispersed in solvent on quartz plates and were allowed to dry at room temperature before anisotropy measurement.

### Fluorescence Anisotropy Measurements

Fluorescence lifetime and anisotropy measurements were performed with Horiba Fluorolog-3 Spectrofluorophotometer with an excitation wavelength at 530 nm. The concentration of **P-ST** and **P-SF** nanoparticles was about 0.5 mg/mL in THF and **P-NP** particles were dispersed in water (0.5 mg/mL).

## Electronic supplementary material

Molecular Organization Induced Anisotropic Properties of Perylene – Silica Hybrid Nanoparticles
