# Supplementary material for: Molecular Organization Induced Anisotropic Properties of Perylene – Silica Hybrid Nanoparticles
Source: Sci Rep. 2017 Aug 10;7:7842. doi: 10.1038/s41598-017-07892-4 (PMC5552683; doi:10.1038/s41598-017-07892-4)
Supplement: Supplementary file 1 — Molecular Organization Induced Anisotropic Properties of Perylene – Silica Hybrid Nanoparticles [file 41598_2017_7892_MOESM1_ESM.pdf]

Supporting Information

Molecular Organization Induced Anisotropic Properties of Perylene – Silica Hybrid Nanoparticles

Deepa Sriramulu,[a] Shuvan Prashant Turaga,[b] Andrew Anthony Bettiol,[b] and Suresh Valiyaveettil,[a]*

[a]Department of Chemistry, National University of Singapore, 3 Science Drive 3, Singapore 117543 [b]Department of Physics, National University of Singapore, 2 Science Drive 3, Singapore 117542

*Email: chmsv@nus.edu.sg

Supplementary method S1. Synthesis of N, N'- bis(3-(triethoxysilyl)propyl)perylene-3,4,9,10-tetracarboxylic acid diimide Precursor (PDI-1).

N, N'- Bis(3-(triethoxysilyl)propyl)perylene-3,4,9,10-tetracarboxylic acid diimide was prepared using a reported procedure. 3,4,9,10-Perylenetetracarboxylic dianhydride (0.392 g, 1 mmol) and APTES (1 mL, 4 mmol) were mixed and stirred under nitrogen atmosphere for 5 min, and refluxed at 130 ºC for 3 h. Dark red colored reaction mixture was cooled to room temperature filtered and solid was washed with hexane to remove excess APTES and dried under vacuum to give red powder. ^{1}H NMR (300 MHz, $CDCl_3$, δ ppm): 8.68 (4H, d), 8.61 (4H, d), 4.2 (4H, t), 3.84 (12H, m), 1.87 (4H, m), 1.25 (18H, t), 0.78 (4 H); MS (APCI-TOF): calculated for $C_{42}H_{50}N_2O_{10}Si_2$: 798.3, found: 799.3 $(M+H)^+$.

Supplementary method S2. Synthesis of N-(2-ethylhexyl)-N'-(3-(triethoxysilyl)propy)perylene-3,4,9,10-tetracarboxylic acid diimide precursor (PDI-2)

N-(2-Ethylhexyl) perylene-3,4,9,10-tetracarboxylic acid-3,4-anhydride-9,10-imide was prepared using a reported procedure. Product obtained was further reacted with excess aminopropyltriethoxy silane under inert atmosphere at 120 ºC for 3 hrs. Product was filtered, washed with excess hexane and dried under vacuum to yield reddish brown solid. ^{1}H NMR (300 MHz, $CDCl_3$, δ ppm): 8.62 (8H, m), 4.22 (4H, m), 3.87 (6H, m), 1.98 (5H, m), 1.58 (23H, m). MS (APCI-TOF): calculated for $C_{41}H_{46}N_2O_7Si$: 706.30, found: 707.31$(M+H)^+$.

Supplementary method S3. Synthesis of perylene silica nanoparticles by stober method (P-ST).

Perylene doped silica nanoparticles were synthesized using Stober method. In general, TEOS, and PDI-1 dissolved in THF (6 mL) were added into a flask containing appropriate amounts of water, ammonia solution and absolute ethanol (Table 4). The reaction mixture was stirred at room temperature for 24 hrs. Pink colored turbid solution was centrifuged and the precipitate obtained was redispersed in fresh ethanol using sonication, the process was repeated until supernatant solution showed no fluorescence when exposed under UV light. Perylene silica nanoparticles

obtained were dried in an oven at 70 ºC before using for full characterization. Compositions of reagents used for preparing silica nanoparticles by Stober method are given in Table S1.

Table S1. Composition to prepare perylene Stober silica nanoparticles (P-ST)

	**Ethanol (mL)**	**Ammonia (mL)**	**Water (mL)**	**PDI silane 10^{-5} mol**	**TEOS mol**	**PDI-silane mol/TEOS mol**
**P-ST1**	30	3	6	1.25	0.018	0.07 %
**P-ST2**	200	15	36	10.01	0.022	0.5 %

Supplementary method S4. Synthesis of perylene silica nanoparticles by nanoprecipitation method (P-NP).

Nanoprecipitation method was employed to synthesize perylene silica nanoparticles from difunctionalized perylene monomers without the addition of other silica sources. In a typical synthesis, 0.02 g PDI-1 dissolved in THF (10 ml) was added dropwise to water (200 mL) containing ammonia solution (1 M) under vigorous stirring for 3 hours. The solution was freeze dried at - 80 ºC and lyophilized to get dark red particles, washed with methanol to remove all unreacted monomers to get P-NP nanoparticles. The particles were fully characterized using a range of techniques.

Supplementary method S5. Synthesis of perylene functionalized silica nanoparticles (P-SF).

A general procedure to synthesize surface functionalized silica nanoparticles is described below. Pure silica nanoparticles were synthesized using water in oil (W/O) microemulsion method.[1] A solution of PDI-2 (0.005 g) in toluene (2 mL) was added to the dispersion of silica nanoparticles in dry toluene (10 mL). The reaction mixture was refluxed under N_2 atmosphere for 24 hr. Orange colored particles obtained were centrifuged, washed repeatedly with ethanol and THF to remove unreacted perylene silane precursor, filtered and dried in an oven at 70 ºC.

1. Bagwe, R. P., Yang, C., Hilliard, L. R. & Tan, W. Optimization of Dye-Doped Silica Nanoparticles Prepared Using a Reverse Microemulsion Method. *Langmuir,* 2004, **20**, 8336-8342)

Table S2. Particle size and zeta potential of perylene silica nanoparticles.

Particles	SEM particle size (nm)	DLS zetasizer (d.nm)	Zeta potential (mV)
**P-ST1**	300	333	-38.5
**P-ST2**	75	233	-42
**P-SF**	50	200	-40
**P-NP**	50-75	101	-30.2

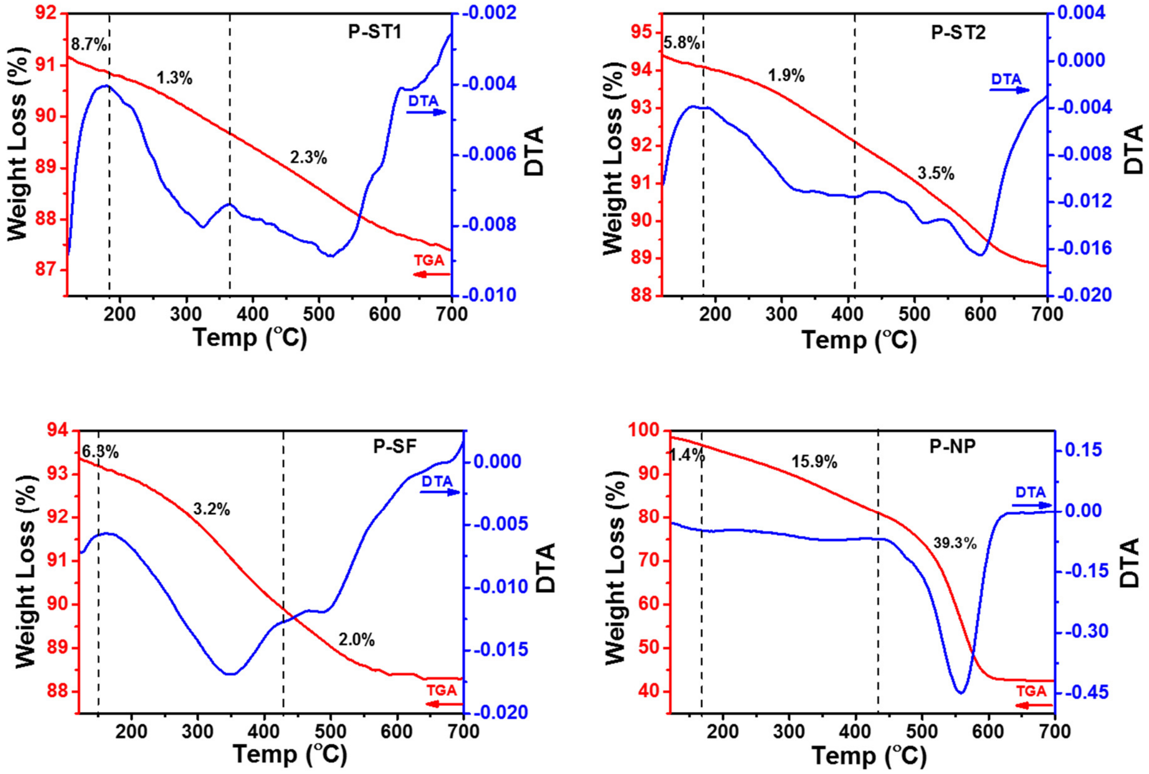

Figure S1. TGA-DTA traces of **P-ST1**, **P-ST2**, **P-SF**, and **P-NP** done in air.

Table S3. Weight loss observed from thermogravimetric analysis of perylene incorporated silica nanoparticles.

Particles	50 °C -150 °C (wt. loss %)	150 °C-400 °C (Wt. loss %)	400 °C-800 °C (Wt. loss %)	EAL analysis C/H/N (wt. %)
**P-ST1**	8.7	1.3	2.3	2.64
**P-ST2**	5.8	1.9	3.5	3.74
**P-SF**	6.3	3.2	2.0	7.7
**P-NP**	1.4	15.9	39.3	43.9

Table S4. Theoretical organic content (wt%) in silica nanoparticles based on the amount of starting materials used for the synthesis

	Total Organic Content in Silica nanoparticles (wt. %)	Loss of alkyl group (wt. %)	Loss Perylene core (wt. %)
**P-ST1**	0.327	0.05	0.26
**P-ST2**	2.13	0.4	1.7
**P-SF**	5.43	1.5	3.8
**P-NP**	59	11	48

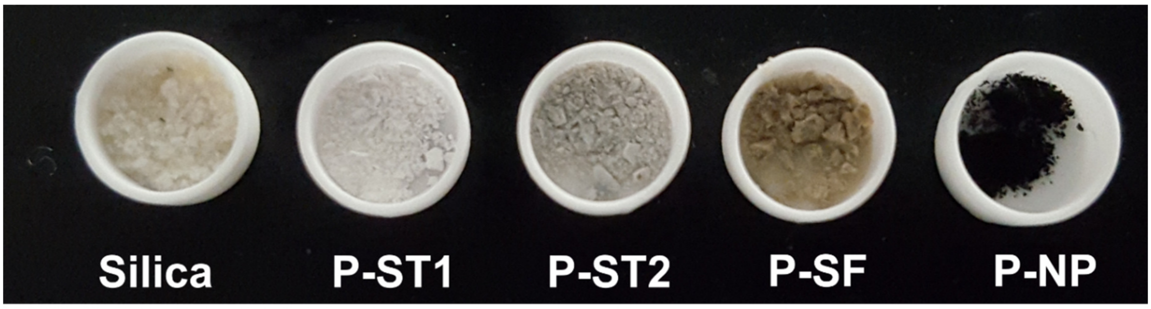

Figure S2. Image of perylene silica nanoparticles after calcinations at 850 °C under nitrogen atmosphere.

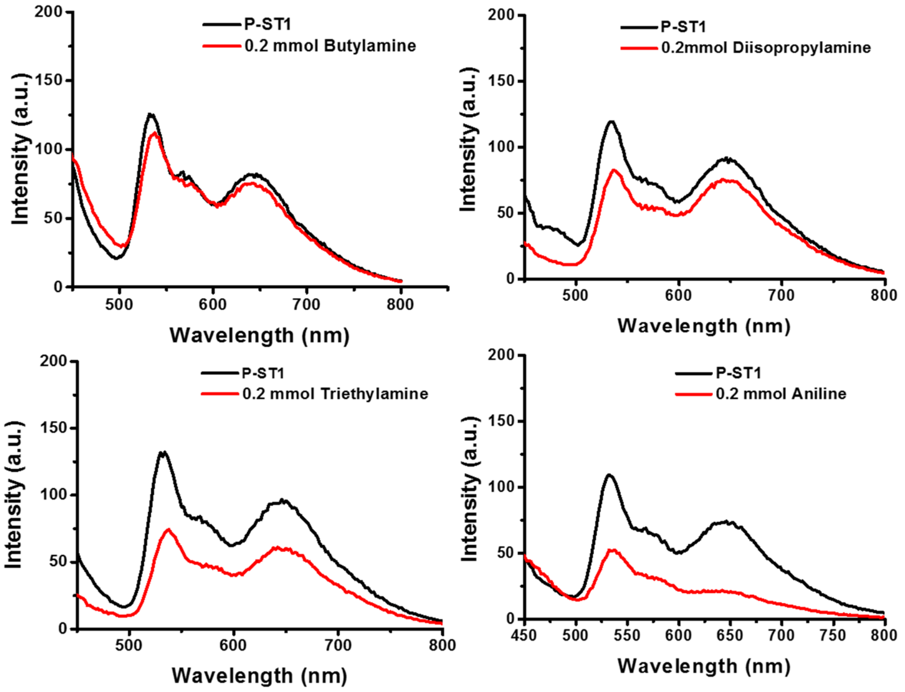

Figure S3. Fluorescence spectra of **P-ST1** perylene silica nanoparticles before and after exposure of amines in THF, (λ_{exi} = 350 nm).

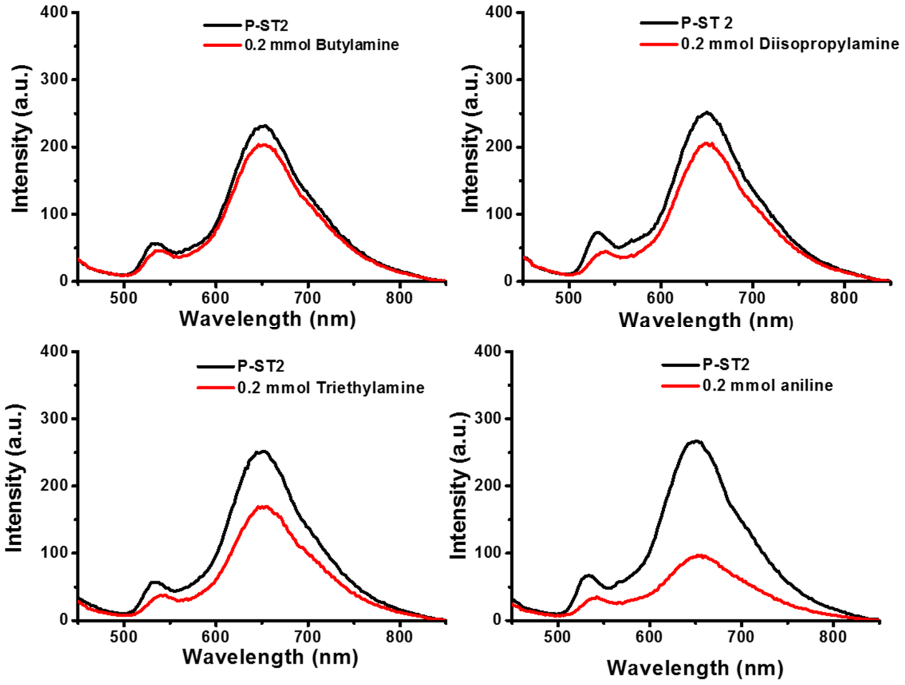

Figure S4. Fluorescence spectra of **P-ST2** perylene silica nanoparticles before and after exposure of amines in THF, (λ_{exi} = 350 nm).

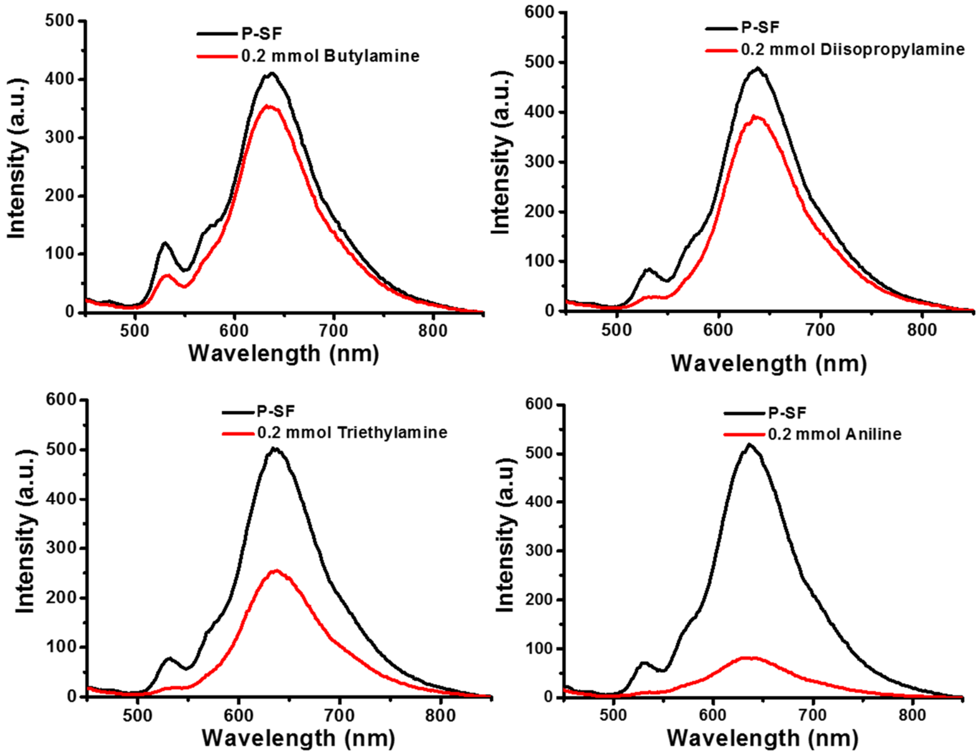

Figure S5. Fluorescence spectra of **P-SF** perylene silica nanoparticles before and after exposure of amines in THF, (λ_{exi} = 350 nm).

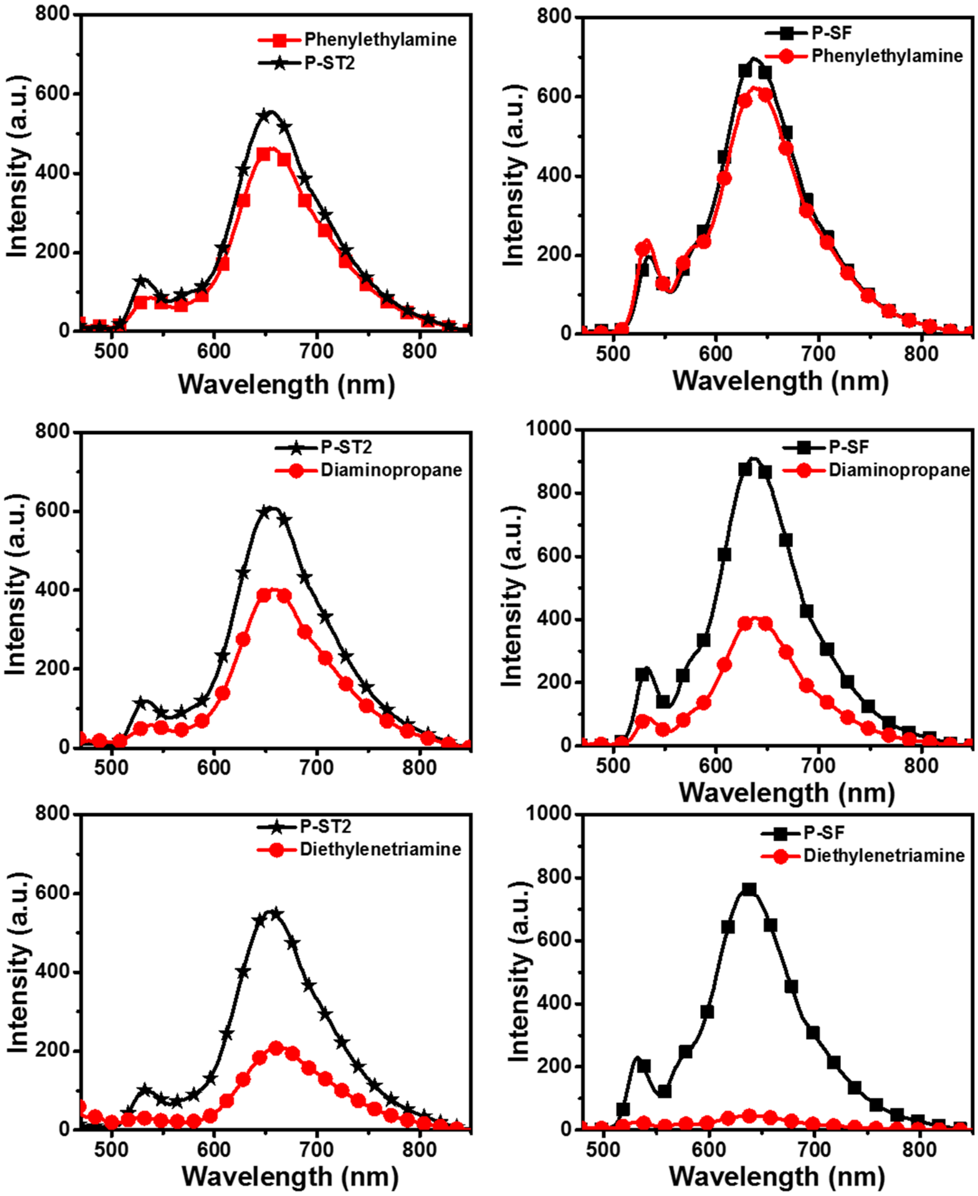

Figure S6. Fluorescence spectra perylene silica nanoparticles (**P-ST2** and **P-SF**) in presence of biogenic amines in solution, (λ_{exi} = 350 nm).

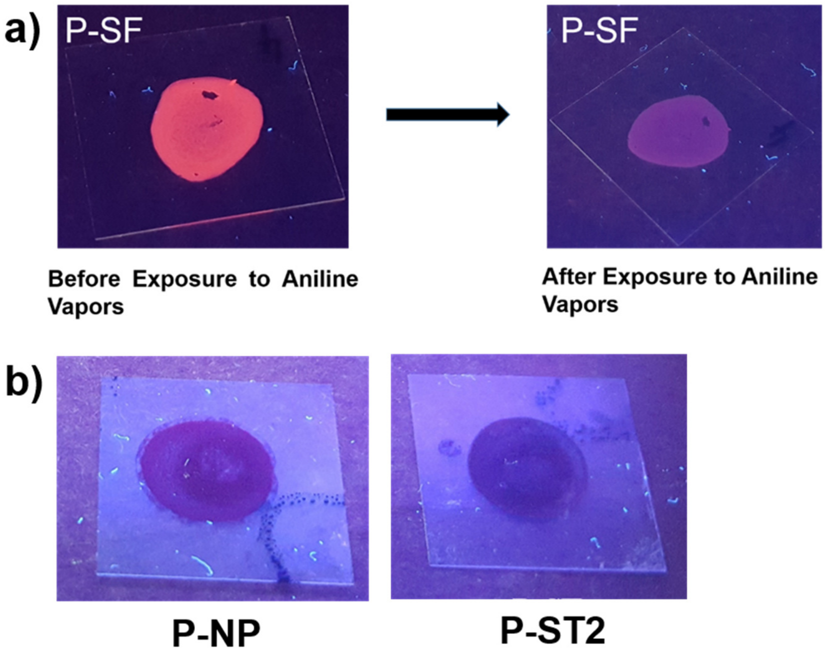

Figure S7. (a) Change in fluorescence intensity of **P-SF** nanoparticles before and after exposure to saturated aniline vapors (15 mins) under the exposure of UV lamp source and (b) **P-NP** and **P-ST2** particles after sensing amine vapors. The films were prepared by dropcasted from dispersion of the particles in THF.

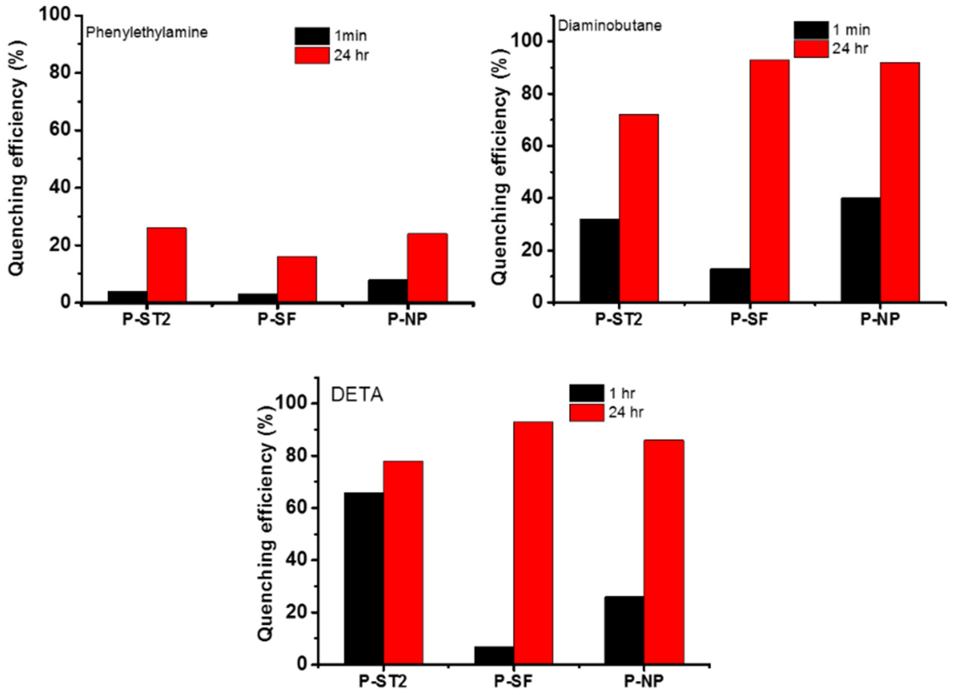

Figure S8. Fluorescence quenching efficiency percentage of perylene silica nanoparticles (P-**ST2**, **P-SF** and **P-NP**) in presence of saturated biogenic amine vapors phenylethylamine (PEA), diaminobutane (DAB) and diethylenetriamie (DETA). (λ_{exi} = 350 nm)
